# Real-World Safety of Vedolizumab in Inflammatory Bowel Disease: A Retrospective Cohort Study Supported by FAERS Signal Analysis

**DOI:** 10.3390/ph18081127

**Published:** 2025-07-28

**Authors:** Bojana Milašinović, Sandra Vezmar Kovačević, Srđan Marković, Marija Jovanović, Tamara Knežević Ivanovski, Đorđe Kralj, Petar Svorcan, Branislava Miljković, Katarina Vučićević

**Affiliations:** 1 Department of Pharmacokinetics and Clinical Pharmacy, Faculty of Pharmacy, University of Belgrade, 11000 Belgrade, Serbia; 2 Department of Gastroenterology and Hepatology, University Hospital Medical Center “Zvezdara”, 11000 Belgrade, Serbia; 3 Faculty of Medicine, University of Belgrade, 11000 Belgrade, Serbia

**Keywords:** safety, exposure-adjusted incidence rates, safety signals, vedolizumab, IBD, monoclonal antibody

## Abstract

**Background/Objectives**: Vedolizumab is a gut-selective anti-integrin monoclonal antibody approved for the treatment of inflammatory bowel disease (IBD). While clinical trials have demonstrated a favorable safety profile, real-world studies are essential for identifying rare adverse events (AEs) and evaluating post-marketing safety. This study assessed vedolizumab’s safety in a real-world cohort and supported the detection of potential safety signals. **Methods**: A retrospective chart review was conducted on adult IBD patients treated with vedolizumab at a tertiary center in the Republic of Serbia between October 2021 and August 2022. Data included demographics, AEs, and newly reported extraintestinal manifestations (EIMs). Exposure-adjusted incidence rates were calculated per 100 patient-years (PYs). Disproportionality analysis using the FDA Adverse Event Reporting System (FAERS) was performed to identify safety signals, employing reporting odds ratios (RORs) and proportional reporting ratios (PRRs) for AEs also observed in the cohort. Prior IBD therapies and reasons for discontinuation were evaluated. **Results**: A total of 107 patients (42.1% Crohn’s disease, 57.9% ulcerative colitis) were included, with a median vedolizumab exposure of 605 days. There were 92 AEs (56.51/100 PYs), most frequently infections (23.95/100 PYs), gastrointestinal disorders (4.30/100 PYs), and skin disorders (4.30/100 PYs). The most frequently reported preferred terms (PTs) included COVID-19, COVID-19 pneumonia, nephrolithiasis, and nasopharyngitis. Arthralgia (12.90/100 PYs) was the most frequent newly reported EIM. No discontinuations due to vedolizumab AEs occurred. FAERS analysis revealed potential signals for events not listed in prescribing information but observed in the cohort: nephrolithiasis, abdominal pain, diarrhea, malaise, cholangitis, gastrointestinal infection, blood pressure decreased, weight decreased, female genital tract fistula, respiratory symptom, and appendicectomy. Most patients had received three prior therapies, often stopping one due to AEs. **Conclusions**: Vedolizumab demonstrated a favorable safety profile in the IBD cohort. However, FAERS-identified signals, such as nephrolithiasis, gastrointestinal infections, and decreased blood pressure, warrant further investigation in larger, more diverse populations.

## 1. Introduction

Inflammatory bowel diseases (IBDs) represent a group of heterogeneous, chronic, idiopathic inflammatory conditions primarily affecting the gastrointestinal tract, of which the principal phenotypes are Crohn’s disease (CD) and ulcerative colitis (UC). IBD is hypothesized to result from an inappropriate immune activation against normally present microorganisms in genetically predisposed individuals [1]. The incidence of IBD increased markedly in Western countries during the previous century and is currently increasing in developing countries as well [2]. With over one million individuals affected in the USA and approximately 2.5 million in Europe, IBD represents a significant public health concern in the 21st century, imposing a considerable burden on healthcare systems [3].

Standard treatment strategies for IBD include aminosalicylates, corticosteroids, immunomodulators, and biologic agents [4]. Although tumor necrosis factor-alpha (TNF-α) inhibitors have revolutionized IBD treatment since the first approval more than 25 years ago, this treatment also has some limitations. More than 30% of patients are primary non-responders, over 40% experience a secondary loss of response, and the treatment is associated with important side effects, including severe infections [5]. These limitations underscore the ongoing need for alternative therapeutic options, such as vedolizumab, a gut-selective integrin antagonist with a distinct mechanism of action [6].

Vedolizumab is a humanized monoclonal IgG1 antibody that targets the α_4_β_7_ integrin expressed on human leukocytes [7]. By blocking the interaction between leukocytes and their ligand, mucosal vascular addressin cell adhesion molecule 1 (MAdCAM-1), on intestinal vascular endothelium, vedolizumab limits intestinal inflammation [8]. Owing to its selectivity, it does not inhibit other integrins, and the α_4_β_1_–vascular cell adhesion protein 1 (VCAM-1) interaction and consequently lymphocyte trafficking to the CNS are unaffected. This results in a significantly lower risk of progressive multifocal leukoencephalopathy (PML) compared to the previously developed anti-integrin agent natalizumab [9]. Based on the results of registration trials, which demonstrated the efficacy and safety of vedolizumab treatment in UC (GEMINI 1) [10] and CD (GEMINI 2 and GEMINI 3) [11,12], vedolizumab was approved by the FDA (Food and Drug Administration) in May 2014 for the treatment of moderately to severely active CD and UC [13].

Vedolizumab is considered to have a favorable safety profile, attributed to its gut-selective mechanism of action, which limits systemic immunosuppression, distinguishing it from other systemic IBD therapies. In the phase 3 GEMINI studies, vedolizumab demonstrated a favorable short-term safety profile, with the most frequently reported adverse events (AEs) being nausea, vomiting, headache, upper respiratory tract infection, arthralgia, nasopharyngitis, and abdominal pain. The overall incidence of AEs was comparable between the vedolizumab and placebo groups [10,11,12]. Long-term vedolizumab safety was assessed in the GEMINI long-term safety (LTS) study, which confirmed its positive benefit-risk profile for the long-term treatment of UC and CD. The most frequently reported AEs during long-term treatment included disease exacerbation, nasopharyngitis, and arthralgia [14]. A combined safety analysis of six clinical trials involving 2932 patients showed a higher exposure-adjusted incidence rate of AEs, serious AEs, and infections in patients receiving placebo compared to those treated with vedolizumab [15].

It is well known that there are significant differences between the clinical trial population and real-world patients, due to strong inclusion and exclusion criteria in clinical trials. Besides that, some rare events and events with longer time-to-onset are usually detected once the drug is on the market, since a significantly higher number of patients are exposed to the drug, with potentially longer exposure. A post-marketing study conducted by Takeda based on data from the global vedolizumab safety database showed a safety profile consistent with the findings from the GEMINI LTS study [16]. Additionally, a systematic review and meta-analysis of observational studies confirmed vedolizumab’s favorable safety profile with low rates of serious infections [17].

Ongoing surveillance of real-world data is of utmost importance for further characterization of the safety profiles of marketed drugs and for evaluation of their long-term safety in clinical practice. The aim of our study was to assess AEs and the safety profile of vedolizumab in IBD patients and to further support the detection of potential safety signals using the FDA Adverse Event Reporting System (FAERS) database.

## 2. Results

A total of 107 patients were included in our analysis (57.9% patients with UC; 50.5% male patients). Median duration of vedolizumab exposure was 605 days, with a total exposure of 162.81 PYs. The majority of patients were previously exposed to immunosuppressives (81.3%) and corticosteroids (75.7%), while almost half (43.0%) had been previously treated with anti-TNF-α drugs. Baseline demographics and clinical characteristics of our patient cohort are presented in more detail in Table 1.

AEs observed in our study are presented per MedDRA PT and SOC in Table 2, with calculated exposure-adjusted incidence rates. A total of 92 AEs (56.51 events/100 PYs) were recorded. The most frequently reported SOCs were Infections and infestations (39 events; 23.95 events/100 PYs), Gastrointestinal disorders (7 events; 4.30/100 PYs), and Skin and subcutaneous tissue disorders (7 events; 4.30 events/100 PYs). At the PT level, the most frequently reported events were COVID-19 (23 events; 14.13/100 PYs), COVID-19 pneumonia (4 events; 2.46/100 PYs), nephrolithiasis (4 events; 2.46/100 PYs), and nasopharyngitis (3 events; 1.84/100 PYs). The following PTs were each reported twice (1.23/100 PYs): peripheral oedema, increased body temperature, erythema, gastrointestinal infection, nausea, and urinary tract infection. All remaining PTs were single occurrences. Importantly, no patient discontinued vedolizumab treatment due to an AE during the study period. Causality assessment between AEs and vedolizumab was not systematically documented in hospital discharge records. In only two patients, it was clearly stated that the AEs were considered related to vedolizumab administration. One patient experienced a drop in blood pressure during vedolizumab administration, and the other one experienced facial eczema after 12 days of the first dose. The eczema resolved completely within five days following treatment with an antihistamine. For all other AEs, it remained unclear if treating physicians attributed them to vedolizumab.

**Figure 1:**
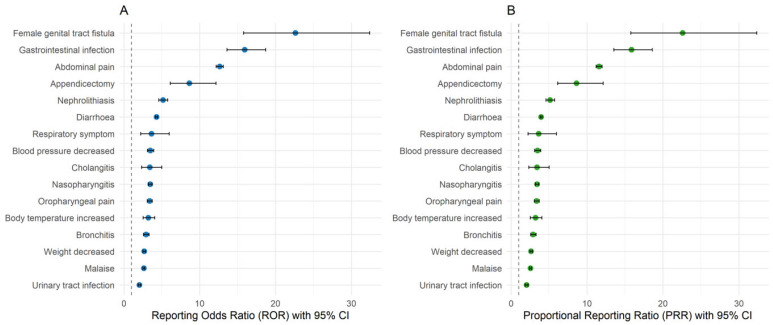
Disproportionality analysis results for selected preferred terms (PTs) from our study that met criteria for signals of disproportionate reporting in the FAERS database. (**A**) Reporting odds ratios (RORs) with 95% confidence intervals (CI) for selected PTs. (**B**) Proportional reporting ratios (PRRs) with 95% CI for the same PTs.

The PTs reported in our study that met the criteria for signals of disproportionate reporting in the FAERS database are presented in Figure 1. Among them, the following PTs were considered unlisted per the current vedolizumab U.S. Prescribing Information (USPI): nephrolithiasis, abdominal pain, diarrhea, malaise, cholangitis, gastrointestinal infection, decreased blood pressure, decreased weight, female genital tract fistula, respiratory symptoms, and appendicectomy. Additional disproportionality analysis results for all PTs reported in our study, along with their listedness per vedolizumab USPI, are provided in Supplementary Table S1.

A total of 28 (17.20/100 PYs) new EIMs were identified following vedolizumab initiation, as shown in Table 3. The most frequently reported new EIM was arthralgia (21 events, 12.90/100 PYs), and this was the only PT among the new EIMs that met the criteria for a signal of disproportionate reporting in the FAERS database, as ROR was 2.776 (95% CI: 2.646, 2.914) and PRR was 2.793 (95% CI: 2.662, 2.931). Arthralgia is a listed event per the current vedolizumab USPI.

**Figure 2:**
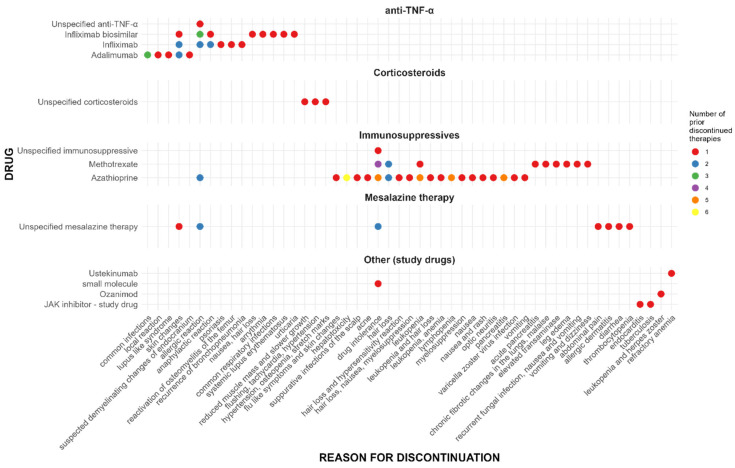
Inflammatory bowel disease (IBD) therapies previously discontinued due to adverse events and corresponding reasons for discontinuation.

A total of 368 therapies for IBD prior to vedolizumab were reported, with a median of 3 therapies per patient (IQR: 2–4). Of these, 197 therapies had been previously discontinued, with a median of 2 discontinued therapies per patient (IQR: 1–3). Among the discontinued treatments, 102 were stopped due to AEs, with a median of 1 therapy per patient (IQR: 0–2). Previously discontinued therapies, along with reasons for discontinuation, are presented in Figure 2. The most frequently discontinued therapies due to AEs were immunosuppressives (*n* = 57). Among these, azathioprine was discontinued in 43 cases, with the most frequently reported reasons being leukopenia and lymphopenia, hepatotoxicity, drug intolerance, and pancreatitis. Methotrexate was discontinued in 13 cases, most commonly due to drug intolerance. The second most commonly discontinued group of drugs was anti-TNF-α inhibitors (*n* = 28). Infliximab biosimilars were discontinued in 10 cases and originator infliximab in 9 cases, most frequently due to allergic reactions. Adalimumab was discontinued in eight cases, primarily due to infections or dermatologic adverse effects. In the remaining cases, mesalazine was discontinued in nine patients, corticosteroids in three patients, and other (study) drugs in five patients.

## 3. Discussion

Our real-world cohort of vedolizumab-treated patients shared several characteristics (presented in Table 1) with those enrolled in clinical trials. For example, the gender distribution was similar, with 50.5% male patients in our study compared to 50% reported in an integrated analysis of clinical trials by Colombel et al. [15]. The average time from IBD diagnosis to vedolizumab initiation was also comparable (9 years in our cohort vs. 8.7 years in Colombel et al.’s analysis). In contrast, our patients were older at treatment initiation (48 years vs. 38.2 years). Fewer patients in our cohort used concomitant corticosteroids (20.6% vs. 53%) or immunosuppressants (24.3% vs. 32%) compared to those in the integrated analysis. Median vedolizumab exposure was longer in our study (605 days) compared to Colombel et al. (378 days), but shorter than in the GEMINI LTS study (42.4 months in UC and 31.5 months in CD patients in LTS) [14]. The proportion of patients with prior anti-TNFα exposure was similar to that in the GEMINI 1 and 2 trials [10,12].

The incidence of AEs reported in our study was substantially lower than that observed in clinical trials (56.51 events/100 PYs vs. 247.8 events/100 PYs in the analysis by Colombel et al.), likely due to the retrospective design of our study. Furthermore, AEs may be underreported in real-world settings when patients are not directly asked about them, and especially when a longer interval has elapsed between the occurrence of the AE and hospitalization. In our study, clinical data were extracted solely from discharge summaries, which may have contributed to incomplete AE capture.

In our study, Infections and infestations represented the most frequently reported SOC, and within this SOC, the most frequently reported AEs at the PT level were COVID-19, COVID-19 pneumonia, and nasopharyngitis, as shown in Table 2. These findings are mostly in line with previous clinical trial data, where respiratory tract infections (RTIs), particularly upper respiratory tract infections (URTIs), were among the most commonly reported AEs in patients treated with vedolizumab. In the GEMINI trials and a subsequent pooled analysis, the incidence of URTIs was numerically higher in the vedolizumab group compared to placebo, although this difference did not reach statistical significance [18]. Specifically, nasopharyngitis occurred more frequently in vedolizumab-treated patients compared to placebo, with some meta-analyses reporting a statistically significant increased risk in CD populations, as RR was 1.77 (95% CI: 1.01, 3.10), *p* = 0.045 [5]. Although previous studies have shown that vedolizumab does not cause systemic immunosuppression due to its gut-selective mechanism of action [7], the observed higher rates of URTIs may be potentially explained by MAdCAM-1 expression in the oropharynx [8]. The relatively high incidence of COVID-19-related events observed in our cohort likely reflects both the temporal overlap of data collection with the COVID-19 pandemic and the heightened susceptibility of IBD patients receiving immunomodulatory therapy to viral infections. Regarding lower respiratory tract infections (LRTIs), the incidence of COVID-19 pneumonia was notable in our study; however, this contrasts with the findings of Feagan et al. [18], where the incidence of LRTIs, including pneumonia, was numerically higher in the placebo group, without reaching statistical significance. Our findings may be partially explained by the established increased risk of pneumonia in patients with IBD itself [19]. Notably, most RTIs reported in clinical trials and in our study were mild or moderate in severity and did not lead to treatment discontinuation. Nonetheless, continued vigilance is warranted, especially during pandemics, given the underlying infection risk in this patient population.

At the SOC level, events classified under Skin and subcutaneous tissue disorders were among the most frequently reported in our study (7 events; 4.30 events/100 PYs), which has not been observed in clinical trials and previous real-world studies. A total of seven events were reported: two cases of erythema and one case each of alopecia, eczema, abnormal hair growth, pruritus, and skin plaque. In addition, gastrointestinal disorders were also among the most frequently reported SOCs (7 events; 4.30 events/100 PYs), which is expected given the underlying IBD in the treated population. However, the observation of skin-related events should be interpreted with caution. Given the small number of events and the possibility of coincidental occurrence, no firm conclusions can be drawn at this stage. Further studies are needed to explore a potential association between vedolizumab and skin-related AEs.

At the PT level, among the most frequently reported events in our study was also nephrolithiasis (4 events; 2.46/100 PYs), which has not been recognized as an adverse effect of vedolizumab in either clinical trials or post-marketing surveillance to date [15,20]. However, a recent cross-sectional study using data from the Northwell Hospital Database supports our observation, concluding that vedolizumab use may be associated with an increased risk of nephrolithiasis [21]. This finding may be confounded by the established association between IBD itself and an increased risk of nephrolithiasis [22].

Per the disproportionality analysis of PTs reported in our study using data from the FAERS database, several PTs met the criteria for signals of disproportionate reporting, as presented in Figure 1. While many of these events have already been discussed above as they were among the most frequently reported in our cohort, the following unlisted events (i.e., not included in the current USPI for vedolizumab) also emerged as potential safety signals: malaise, cholangitis, gastrointestinal infection, decreased blood pressure, weight loss, female genital tract fistula, respiratory symptoms, and appendicectomy. Some of these findings are confounded and potentially caused by the underlying IBD, such as malaise, cholangitis, weight loss, and female genital tract fistula. The signal of respiratory symptoms, which was not further specified, may be related to URTIs already discussed above.

Decreased blood pressure was reported in our study as an infusion-related reaction, occurring shortly after vedolizumab infusion. However, we were unable to identify similar findings in the available literature. A possible confounder of this signal could be dehydration due to IBD-associated diarrhea. Therefore, the signal of decreased blood pressure should be closely monitored in future real-world studies.

Appendicectomy was identified as a signal in the disproportionality analysis, but its clinical relevance remains unclear. Given the lack of an established association between vedolizumab and appendicitis, as well as evidence suggesting that the occurrence of appendicitis is not increased in patients with IBD [23], this finding may be incidental. Further data are needed to determine whether this represents a true safety signal or a coincidental procedural event.

The signal of gastrointestinal infection in the FAERS database, along with two reported gastrointestinal infections (1.23/100 PYs) in our study, warrants further discussion. This is supported by previous integrated analysis of clinical trials showing a higher, although statistically insignificant, incidence of gastrointestinal infections in the vedolizumab group compared to the placebo (7.4/100 PYs vs. 6.7/100 PYs) and an analysis of six real-world cohort studies showing a rate of enteric infection of 2% (21/1049) [24]. This signal may be biologically plausible given vedolizumab’s gut-selective mechanism of action and consequent localized suppression of immune responses in the gut [14]. Ongoing monitoring is needed to further assess this potential risk.

These results emphasize the need for safety monitoring and continued pharmacovigilance during vedolizumab therapy. While the identified signals, such as nephrolithiasis, gastrointestinal infections, and infusion-related hypotension, require cautious interpretation, they highlight potential areas of clinical relevance. In practice, clinicians should remain vigilant for such adverse events, especially in patients with underlying risk factors or complex treatment histories, to support early detection and appropriate treatment.

Our finding that the most frequently reported EIM was arthralgia (21 events, 12.90/100 PYs), which also met the criteria for a signal of disproportionate reporting in the FAERS database, is consistent with previous studies, real-world data, and vedolizumab USPI [14,20,25]. No other EIMs emerged as potential safety signals in our analysis.

Notably, no new malignancies emerged in our cohort, aligning with prior findings on the safety of vedolizumab regarding malignancy risk [26]. Additionally, five patients in our cohort had a history of carcinoma prior to vedolizumab initiation, which aligns with studies suggesting that vedolizumab does not increase the risk of cancer recurrence in patients with prior malignancy [27]. However, the follow-up period in our study is relatively short and may be insufficient to assess long-term malignancy risk. We were unable to assess the risk of postoperative complications, an area with conflicting findings in previous studies and meta-analyses [28,29], as only one patient underwent surgery during our study period.

Information on prior IBD therapies in our patient cohort provides important context for interpreting vedolizumab safety data. This was a heavily pre-treated population, with a median of three prior therapies and two discontinued therapies per patient before study enrollment. On average, each patient had discontinued one previous treatment due to an AE. Most AEs that led to discontinuation of prior treatments were already known side effects of those therapies, as shown in Figure 2. This is noteworthy given that no patients in our study discontinued vedolizumab due to an AE during the study period. From a clinical perspective, this finding further supports the favorable safety profile of vedolizumab, particularly in a population with significant prior treatment exposure and a history of intolerance to other therapies. The low rate of treatment discontinuation due to AEs suggests that vedolizumab may be a well-tolerated option for long-term treatment of moderate-to-severe IBD. Clinicians may consider this data when selecting therapy for patients with complex treatment histories, intolerance to other agents, or increased susceptibility to systemic side effects.

There are several limitations to our study that should be considered. It was retrospective and conducted at a single tertiary center, which may limit how applicable the findings are to other populations. Due to its retrospective nature, the study is subject to limitations in data consistency and completeness. Specifically, AEs were collected from discharge summaries rather than through a structured reporting system, so some events may have been missed or not fully described. Because there was no systematic collection of AEs, causality and seriousness assessments were not routinely performed or documented by treating physicians. Moreover, during the retrospective review, the available information was often insufficient to appropriately assess event seriousness and causality, so these assessments were not performed retrospectively. Additionally, dosing information and stratification by treatment phase (induction vs. maintenance) were not included in the analysis, which limits the ability to evaluate whether adverse events and safety signals vary by treatment phase and reduces comparability of exposure-adjusted incident rates with findings from clinical trials and other real-world studies. In addition, the potential confounding effect of concomitant immunosuppressive therapies, such as corticosteroids or immunomodulators, cannot be excluded, particularly with respect to infections. This potential confounding effect was not adjusted for in our analysis and should be considered when interpreting results. Moreover, we acknowledge the limitation of missing precise time-to-onset data; however, upon review, only two adverse events—eczema and hepatic cytolysis—were documented within the first two months after vedolizumab initiation. Finally, the signals identified through the FAERS database should be interpreted with caution, as this system has limitations common to pharmacovigilance databases. As a spontaneous reporting database, FAERS is subject to significant underreporting, as well as selective or delayed reporting. The quality of reports can vary widely, as many cases do not include complete clinical information. Most importantly, spontaneous reporting systems do not contain denominator data, so it is not possible to calculate incidence rates. Therefore, disproportionality signals from FAERS should be interpreted with caution, as detected signals may be related to the underlying disease, comorbidities, or reporting biases rather than to the drug of interest.

## 4. Materials and Methods

A retrospective review of hospital records was conducted for patients with UC or CD who were treated with vedolizumab at the University Medical Center “Zvezdara” in Belgrade, Republic of Serbia, between October 2021 and August 2022. The study protocol was approved by the Institutional Ethics Committee (No. IRB00009457, on 5 October 2022). A total of 107 adult (aged ≥ 18 years) patients who received vedolizumab during the induction and maintenance phase were enrolled. Baseline patient characteristics collected for this study included: age at vedolizumab initiation, gender, underlying diagnosis (UC or CD), age at diagnosis confirmation, presence of extraintestinal manifestations (EIMs) at vedolizumab initiation, number of comorbidities, prior therapies (immunosuppressives, corticosteroids, and TNF-α inhibitors), and concomitant therapies (corticosteroids and/or immunosuppressives). It is worth mentioning that pharmacokinetic parameters and predictors for clinical and endoscopic remission for this patient cohort were previously analyzed and published [30].

According to pharmacovigilance definitions, an AE is considered to be any untoward medical occurrence, irrespective of its relatedness to the drug, whereas an adverse reaction refers specifically to an AE considered to be drug-related [31]. All AEs, regardless of causality, were collected throughout vedolizumab exposure for all enrolled patients. Symptoms of progression of underlying CD or UC were not considered to be AEs for the purpose of this study and were not collected as such. All new EIMs that occurred after vedolizumab initiation and were not present at baseline were collected. Exposure to vedolizumab was calculated for each patient as the time from the first dose to either the date of vedolizumab discontinuation or the date of the last available hospital discharge, whichever occurred first. Exposure-adjusted incidence rates were calculated for all AEs and EIMs and expressed as the number of patients who experience the event per 100 person–years (PYs) of exposure [32].

All AEs and new EIMs were coded using the Medical Dictionary for Regulatory Activities (MedDRA), a standardized medical terminology developed by the International Council for Harmonization (ICH) and maintained by Maintenance and Support Services Organization (MSSO) [33]. MedDRA has a hierarchical structure, with lowest level terms (LLTs) being at the bottom of the hierarchy, followed by preferred terms (PTs), high level terms (HLTs), high level group terms (HLGTs), and finally system organ classes (SOCs). MedDRA is updated twice annually, in March and September. For the purpose of this study, MedDRA version 27.1 (released in September 2024) was effective and used for the coding. Additionally, for each AE, it was assessed if this is a labeled event, based on the effective labeling document [20].

To determine if any of the AEs or new EIMs observed in our study might represent a potential safety signal, we performed a disproportionality analysis using the FAERS database. FAERS is a repository of AEs and medication errors reported to the FDA, aimed at supporting post-marketing safety surveillance [34]. We utilized OpenVigil 2.1 [35], an open-access tool for data mining and analysis of pharmacovigilance data, to perform the disproportionality analysis. Disproportionality analysis was performed to assess if any of the reported AEs on the PT level were disproportionately highly represented in the FAERS database [36].

Reports concerning vedolizumab were included, the role of the drug was selected as ‘primary suspect’, and the time period was defined from 1 January 2014 to 31 December 2024, taking into consideration the vedolizumab approval date. The entire FAERS database for the above-specified time period was used as a background, without any restrictions. We implemented frequentist methods (proportional reporting ratio (PRR) and reporting odds ratio (ROR)) to calculate disproportionality [37]. Frequentist methods are based on 2 × 2 contingency tables that compare the observed number of a drug–AE combination of interest to all other drugs and events in the database [37]. Formulas and pre-defined thresholds for both used measures are presented in Table 4 [37]. Disproportionality values were calculated for all events at the PT level of the MedDRA hierarchy. A drug-event combination was considered a signal of disproportionate reporting (SDR) if it exceeded the thresholds for both PRR and ROR.

In addition, previous therapies for IBD were evaluated for all enrolled patients. This assessment included the average number of previous therapies, the average number of therapies discontinued for any reason, and the average number of therapies discontinued due to an AE. AEs that led to the discontinuation of previous IBD treatments were presented separately.

## 5. Conclusions

This retrospective, single-center study did not find any new safety concerns associated with vedolizumab that would change its known benefit–risk profile in patients with IBD. Nevertheless, a variety of AEs were observed; most were mild, and some were consistent with prior clinical trial results, but did not result in treatment discontinuation. Signals of disproportionate reporting identified through FAERS analysis were noted in our study, such as nephrolithiasis, gastrointestinal infections, and decreased blood pressure. In the context of prior exposure to multiple IBD therapies, vedolizumab demonstrated a favorable safety profile and emerged as a safer and better-tolerated option compared to previous treatments received by patients in our study. However, the retrospective design, limited causality assessment, and inherent constraints of spontaneous reporting systems should be considered when interpreting these results. Larger prospective, multicenter studies, along with continued post-marketing surveillance and pharmacogenomic research, are needed to confirm these observations, validate potential safety signals, and better understand individual risk factors for adverse events.

## Figures and Tables

**Table 1:** Demographics and clinical characteristics of our cohort of patients at baseline (*n* = 107).

Variable	Value
Age at time of vedolizumab initiation, median (IQR)	48 (33.5–66)
≥65 years of age, *n* (%)	29 (27.1)
Male gender, *n* (%)	54 (50.5)
CD, *n* (%)	45 (42.1)
UC, *n* (%)	62 (57.9)
EIM present at baseline, *n* (%)	42 (39.2)
Age at time of IBD diagnosis, median (IQR)	38 (22–53)
Years from diagnosis to vedolizumab initiation, median (IQR)	9 (4–17)
Vedolizumab exposure (days), median (IQR)	605 (209–860)
Prior therapy	
Previous immunosuppressive therapy, *n* (%)	87 (81.3)
Previous exposure to anti-TNF-α agents, *n* (%)	46 (43.0)
Previous corticosteroid therapy, *n* (%)	81 (75.7)
Concomitant therapy at baseline	
Concomitant corticosteroids, *n* (%)	22 (20.6)
Concomitant immunosuppressive therapy, *n* (%)	26 (24.3)
Concomitant corticosteroids and immunosuppressive therapy, *n* (%)	5 (4.7)
Number of comorbidities per patient, median (IQR)	1 (0–2)
Age at time of vedolizumab initiation, median (IQR)	48 (33.5–66)

CD: Crohn’s disease; EIM: extraintestinal manifestation; IBD: inflammatory bowel disease; IQR: interquartile range; *n*: number; TNF: tumor necrosis factor; UC: ulcerative colitis.

**Table 2:** Adverse events presented per MedDRA SOC and PT, with exposure-adjusted incidence rates per 100 PYs.

MedDRA SOC/MedDRA PT	Count of MedDRA PT	Incidence/100 PYs
**Renal and urinary disorders**	**4**	**2.46**
Nephrolithiasis	4	2.46
**Blood and lymphatic system disorders**	**1**	**0.61**
Thrombocytopenia	1	0.61
**Cardiac disorders**	**1**	**0.61**
Cardiac failure	1	0.61
**Gastrointestinal disorders**	**7**	**4.30**
Abdominal pain	1	0.61
Coeliac artery stenosis	1	0.61
Diarrhea	1	0.61
Nausea	2	1.23
Stomatitis	1	0.61
Vomiting	1	0.61
**General disorders and administration site conditions**	**5**	**3.07**
Malaise	1	0.61
Oedema peripheral	2	1.23
Asthenia	1	0.61
Chest pain	1	0.61
**Hepatobiliary disorders**	**2**	**1.23**
Cholangitis	1	0.61
Hepatic cytolysis	1	0.61
Immune system disorders	2	1.23
Drug hypersensitivity	1	0.61
Hypersensitivity	1	0.61
**Infections and infestations**	**39**	**23.95**
Bronchitis	1	0.61
Conjunctivitis	1	0.61
COVID-19	23	14.13
COVID-19 pneumonia	4	2.46
Gastrointestinal infection	2	1.23
Herpes zoster	1	0.61
Nasopharyngitis	3	1.84
Sialadenitis	1	0.61
Upper respiratory tract infection	1	0.61
Urinary tract infection	2	1.23
**Injury, poisoning, and procedural complications**	**3**	**1.84**
Exposure during pregnancy	1	0.61
Fall	1	0.61
Joint injury	1	0.61
**Investigations**	**6**	**3.69**
Blood alkaline phosphatase increased	1	0.61
Blood creatinine increased	1	0.61
Blood pressure decreased	1	0.61
Body temperature increased	2	1.23
Weight decreased	1	0.61
**Metabolism and nutrition disorders**	**2**	**1.23**
Hyperlipidemia	1	0.61
Hyperproteinemia	1	0.61
**Neoplasms benign, malignant, and unspecified (incl cysts and polyps)**	**1**	**0.61**
Lipoma	1	0.61
**Nervous system disorders**	**4**	**2.46**
Cerebral small vessel ischemic disease	1	0.61
Paresthesia	1	0.61
Sciatica	1	0.61
Tremor	1	0.61
**Reproductive system and breast disorders**	**2**	**1.23**
Female genital tract fistula	1	0.61
Hematospermia	1	0.61
**Respiratory, thoracic, and mediastinal disorders**	**4**	**2.46**
Oropharyngeal pain	1	0.61
Pleural effusion	1	0.61
Respiratory failure	1	0.61
Respiratory symptom	1	0.61
**Skin and subcutaneous tissue disorders**	**7**	**4.30**
Alopecia	1	0.61
Eczema	1	0.61
Erythema	2	1.23
Hair growth abnormal	1	0.61
Pruritus	1	0.61
Skin plaque	1	0.61
**Surgical and medical procedures**	**1**	**0.61**
Appendicectomy	1	0.61
**Vascular disorders**	**1**	**0.61**
Thrombosis	1	0.61
**Total**	**92**	**56.51**

MedDRA: medical dictionary for regulatory activities; PT: preferred term; PYs: person-years; SOC: system organ class.

**Table 3:** New extraintestinal manifestation (EIM) after vedolizumab exposure, with corresponding disproportionality measures from FAERS data.

	Study Data		FAERS Data				
MedDRA PT	n	Incidence/100 PYs	N	ROR (95% CI)	PRR (95% CI)	Chi Square	Listedness per USPI
Anemia	6	3.68	382	1.465 (1.326, 1.620) *	1.468 (1.328, 1.623)	56.606	Unlisted
Iridocyclitis	1	0.61	6	1.063 (0.477, 2.370)	1.063 (0.477, 2.37)	0.004	Unlisted
Arthralgia **	21	12.90	1587	2.776 (2.646, 2.914) *	2.793 (2.662, 2.931) *	1838.365	Listed
Total	28	17.20					

* Indicates that either ROR or PRR meets the statistical threshold for a signal; ** Indicates MedDRA PTs meeting criteria for a safety signal (i.e., both ROR and PRR meet statistical thresholds). CI: confidence interval; FAERS: FDA adverse event reporting system; MedDRA: medical dictionary for regulatory activities; PRR: proportional reporting ratio; PT: preferred term; PYs: person-years; ROR: reporting odds ratio; USPI: U.S. Prescribing Information.

**Table 4:** Formulas and pre-defined thresholds for measures of disproportionality [37].

Measure of Disproportionality	Formula for Calculation	Statistical Threshold for SDR
Reporting Odds Ratio (ROR)	$ROR=\frac{a\cdot d}{b\cdot c}$	lower bound 95% CI > 1; number of reports ≥ 3
Proportional Reporting Ratio (PRR)	$PRR=\frac{a\cdot (c+d)}{c\cdot (a+b)}$	PRR ≥ 2; Chi-square ≥ 4; number of reports ≥ 3

CI: confidence interval; SDR: signal of disproportionate reporting; a: the observed number of reports of drug of interest and adverse event of interest; b: the number of reports of other adverse events attributed to drug of interest (except the event of interest); c: the number of reports describing the adverse event of interest for all other drugs in the database (except drug of interest); d: the number of reports attributed to all other medications and other adverse events in the database.

## Data Availability

Clinical data that support the findings of this study are not openly accessible due to ethical restrictions. The FDA Adverse Event Reporting System (FAERS) database and source are freely available: openFDA is freely accessible at https://api.fda.gov/drug/event.json, accessed on 1 May 2025. OpenVigil FDA can be used or downloaded at http://openvigil.sourceforge.net, accessed on 1 May 2025.

## Supplementary Materials

The following supporting information can be downloaded at: https://www.mdpi.com/article/10.3390/ph18081127/s1, Table S1: Disproportionality analysis results for selected adverse events, and their listedness per vedolizumab U.S. Prescribing Information (USPI).

## Abbreviations

The following abbreviations are used in this manuscript:

AE	Adverse event
CD	Crohn’s disease
CI	Confidence interval
EIM	Extraintestinal manifestation
FAERS	FDA Adverse Event Reporting System
HLGT	High level group term
HLT	High level term
IBD	Inflammatory bowel disease
ICH	International Council for Harmonization
IQR	Interquartile ratio
LLT	Lowest level term
LTS	Long-term safety
MAdCAM-1	Mucosal vascular addressin cell adhesion molecule 1
MedDRA	Medical Dictionary for Regulatory Activities
MSSO	Maintenance and support services organization
PML	Progressive multifocal leukoencephalopathy
PRR	Proportional reporting ratio
PT	Preferred term
PYs	Patient-years
ROR	Reporting odds ratio
RTI	Respiratory tract infections
SDR	Signal of disproportionate reporting
SOC	System organ class
TNF-α	Tumor necrosis factor-alpha
UC	Ulcerative colitis
URTI	Upper respiratory tract infections
USPI	U.S. Prescribing Information
VCAM-1	Vascular cell adhesion protein 1
