# Supplementary material for: Real-World Safety of Vedolizumab in Inflammatory Bowel Disease: A Retrospective Cohort Study Supported by FAERS Signal Analysis
Source: Pharmaceuticals (Basel). 2025 Jul 28;18(8):1127. doi: 10.3390/ph18081127 (PMC12389730; doi:10.3390/ph18081127)
Supplement: Supplementary file 1 [file pharmaceuticals-18-01127-s001.zip › pharmaceuticals-3733609-supplementary.pdf]

Supplementary Material

Real-World Safety of Vedolizumab in Inflammatory Bowel Disease: A Retrospective Cohort Study Supported by FAERS Signal Analysis

Bojana Milašinović [1], Sandra Vezmar Kovačević[1], Srđan Marković [2,3], Marija Jovanović [1], Tamara Knežević Ivanovski [2], Đorđe Kralj [2], Petar Svorcan [2,3], Branislava Miljković [1], Katarina Vučićević [1,*]

[1]Department of Pharmacokinetics and Clinical Pharmacy, Faculty of Pharmacy, University of Belgrade, 11000 Belgrade, Republic of Serbia

[2]Department of Gastroenterology and Hepatology, University Hospital Medical Center "Zvezdara", 11000 Belgrade, Republic of Serbia

[3] Faculty of Medicine, University of Belgrade, 11000 Belgrade, Republic of Serbia

*Correspondence: katarina.vucicevic@pharmacy.bg.ac.rs

Table S1. Disproportionality analysis results for selected adverse events, and their listedness per vedolizumab U.S. Prescribing Information (USPI).

MedDRA SOC / MedDRA PT	*n*	ROR (95% CI)	PRR (95% CI)	Chi square	Listedness per USPI
**Renal and urinary disorders**					
Nephrolithiasis**	312	5.162 (4.613, 5.777)*	5.125 (4.585, 5.729)*	1016.032	Unlisted
**Blood and lymphatic system disorders**					
Thrombocytopenia	26	0.181 (0.123, 0.265)	0.18 (0.122, 0.264)	96.202	Unlisted
**Cardiac disorders**					
Cardiac failure	59	0.528 (0.409, 0.682)	0.529 (0.41, 0.682)	24.352	Unlisted
**Gastrointestinal disorders**					
Abdominal pain**	3143	12.640 (12.177, 13.12)*	11.589 (11.201, 11.99)*	29493.258	Unlisted
Coeliac artery stenosis	0	0	0	1.509	Unlisted
Diarrhoea**	3623	4.305	3.961	8127.397	Unlisted

		(4.159, 4.457)*	(3.84, 4.086)*		
Nausea	1327	1.238 (1.172, 1.308)*	1.229 (1.166, 1.296)	58.003	Listed
Stomatitis	64	0.777 (0.608, 0.994)	0.778 (0.609, 0.994)	3.847	Unlisted
Vomiting	909	1.501 (1.405, 1.604)*	1.488 (1.395, 1.587)	146.878	Unlisted
**General disorders and administration site conditions**					
Malaise**	1687	2.632 (2.506, 2.764)*	2.553 (2.436, 2.675)*	1.608.906	Unlisted
Oedema peripheral	119	1.12 (0.935, 1.341)	1.119 (0.935, 1.34)	1.393	Unlisted
Asthenia	836	1.778 (1.66, 1.905)*	1.759 (1.645, 1.881)	275.306	Unlisted
Chest pain	234	1.122 (0.986, 1.276)	1.121 (0.986, 1.274)	2.937	Unlisted
**Hepatobiliary disorders**					
Cholangitis**	27	3.425 (2.344, 5.006)*	3.423 (2.343, 5.002)*	43.426	Unlisted
Hepatic cytolysis	8	0.362 (0.181, 0.723)	0.362 (0.181, 0.724)	8.376	Listed
**Immune system disorders**					
Drug hypersensitivity	172	0.408 (0.351, 0.474)	0.411 (0.354, 0.477)	146.518	Listed
Hypersensitivity	122	0.459 (0.384, 0.548)	0.461 (0.386, 0.55)	76.822	Listed
**Infections and infestations**					
Bronchitis**	253	2.926 (2.584, 3.314)*	2.912 (2.574, 3.295)*	313.493	Listed
Conjunctivitis	24	0.855 (0.573, 1.276)	0.855 (0.573, 1.276)	0.454	Unlisted
COVID-19	234	1.488	1.485	36.577	Unlisted

		(1.308, 1.693)*	(1.307, 1.688)		
COVID-19 pneumonia	6	0.535 (0.24, 1.192)	0.535 (0.24, 1.192)	1.978	Unlisted
Gastrointestinal infection**	161	15.937 (13.596, 18.682)*	15.868 (13.546, 18.588)*	2115.483	Unlisted
Herpes zoster	137	1.782 (1.506, 2.109)*	1.779 (1.504, 2.104)	45.783	Unlisted
Nasopharyngitis**	882	3.485 (3.259, 3.728)*	3.422 (3.205, 3.654)*	1503.709	Listed
Sialoadenitis	3	1.627 (0.523, 5.059)	1.626 (0.523, 5.059)	0.231	Unlisted
Upper respiratory tract infection	72	1.25 (0.991, 1.575)	1.249 (0.991, 1.574)	3.321	Listed
Urinary tract infection**	451	2.048 (1.866, 2.248)*	2.035 (1.856, 2.231)*	236.171	Listed
**Injury, poisoning and procedural complications**					
Exposure during pregnancy	36	0.321 (0.231, 0.445)	0.321 (0.232, 0.445)	51.035	Unlisted
Fall	546	1.311 (1.204, 1.427)*	1.306 (1.202, 1.42)	39.145	Unlisted
Joint injury	48	1.89 (1.423, 2.511)*	1.889 (1.422, 2.508)	19.091	Unlisted
**Investigations**					
Blood alkaline phosphatase increased	14	0.621 (0.368, 1.05)	0.621 (0.368, 1.05)	2.856	Unlisted
Blood creatine increased	4	0.683 (0.256, 1.822)	0.683 (0.256, 1.822)	0.313	Unlisted
Blood pressure decreased**	283	3.503 (3.114, 3.941)*	3.483 (3.099, 3.914)*	493.839	Unlisted
Body temperature increased**	70	3.219 (2.543, 4.075)*	3.215 (2.541, 4.067)*	103.526	Listed
Weight decreased**	929	2.681	2.636 (2.473, 2.81)*	943.033	Unlisted

		(2.511, 2.862)*			
**Metabolism and nutrition disorders**					
Hyperlipidaemia	8	0.834 (0.417, 1.67)	0.835 (0.417, 1.67)	0.122	Unlisted
Hyperproteinaemia	0	0	0	0.135	Unlisted
**Neoplasms benign, malignant and unspecified (including cysts and polyps)**					
Lipoma	5	1.780 (0.739, 4.289)	1.780 (0.739, 4.288)	1.010	Unlisted
**Nervous system disorders**					
Cerebral small vessel ischaemic disease	0	0	0	0.014	Unlisted
Paraesthesia	161	0.774 (0.663, 0.904)	0.775 (0.664, 0.904)	10.345	Unlisted
Sciatica	30	1.623 (1.133, 2.323)*	1.622 (1.133, 2.322)	6.514	Unlisted
Tremor	98	0.465 (0.381, 0.566)	0.466 (0.382, 0.568)	59.692	Unlisted
**Reproductive system and breast disorders**					
Female genital tract fistula**	32	22.615 (15.784, 32.402)*	22.595 (15.775, 32.363)*	593.764	Unlisted
Haematospermia	1	1.128 (0.158, 8.035)	1.128 (0.158, 8.035)	0.170	Unlisted
**Respiratory, thoracic and mediastinal disorders**					
Oropharyngeal pain**	620	3.402 (3.088, 3.748)*	3.373 (3.066, 3.711)*	693.458	Listed
Pleural effusion	39	0.530 (0.387, 0.725)	0.530 (0.387, 0.726)	15.767	Unlisted
Respiratory failure	19	0.217 (0.138, 0.34)	0.217 (0.139, 0.341)	52.914	Unlisted
Respiratory symptom**	16	3.651 (2.23, 5.979)*	3.650 (2.23, 5.975)*	27.843	Unlisted
**Skin and subcutaneous tissue disorders**					

Alopecia	298	0.962 (0.858, 1.078)	0.962 (0.859, 1.077)	0.415	Unlisted
Eczema	60	0.822 (0.638, 1.06)	0.823 (0.639, 1.06)	2.117	Unlisted
Erythema	168	0.766 (0.658, 0.892)	0.767 (0.66, 0.892)	11.664	Unlisted
Hair growth abnormal	0	0	0	10.429	Unlisted
Pruritus	362	0.661 (0.595, 0.733)	0.664 (0.599, 0.736)	62.02	Listed
Skin plaque	7	0.470 (0.224, 0.986)	0.470 (0.224, 0.986)	3.667	Unlisted
**Surgical and medical procedures**					
Appendicectomy**	34	8.633 (6.138, 12.143)*	8.625 (6.134, 12.128)*	215.381	Unlisted
**Vascular disorders**					
Thrombosis	145	1.29 (1.095, 1.519)*	1.288 (1.095, 1.516)	9.059	Unlisted

* Indicates that either ROR or PRR meets the statistical threshold for a signal; ** Indicates MedDRA PTs meeting criteria for a safety signal (i.e., both ROR and PRR meet statistical thresholds).

MedDRA: medical dictionary for regulatory activities; *n*: number; PRR: proportional reporting ratio; PT: preferred term; ROR: reporting odds ratio; SOC: system organ class; USPI: U.S. prescribing information.
